# A unified model for yeast transcript definition

**DOI:** 10.1101/gr.164327.113

**Published:** 2014-01

**Authors:** Carl G. de Boer, Harm van Bakel, Kyle Tsui, Joyce Li, Quaid D. Morris, Corey Nislow, Jack F. Greenblatt, Timothy R. Hughes

**Affiliations:** 1 Department of Molecular Genetics,; 2 Banting and Best Department of Medical Research and Donnelly Centre for Cellular and Biomolecular Research,; 3 Department of Pharmaceutical Sciences, University of Toronto, Toronto, Ontario, M5S 3E1, Canada

## Abstract

Identifying genes in the genomic context is central to a cell's ability to interpret the genome. Yet, in general, the signals used to define eukaryotic genes are poorly described. Here, we derived simple classifiers that identify where transcription will initiate and terminate using nucleic acid sequence features detectable by the yeast cell, which we integrate into a Unified Model (UM) that models transcription as a whole. The *cis*-elements that denote where transcription initiates function primarily through nucleosome depletion, and, using a synthetic promoter system, we show that most of these elements are sufficient to initiate transcription in vivo. Hrp1 binding sites are the major characteristic of terminators; these binding sites are often clustered in terminator regions and can terminate transcription bidirectionally. The UM predicts global transcript structure by modeling transcription of the genome using a hidden Markov model whose emissions are the outputs of the initiation and termination classifiers. We validated the novel predictions of the UM with available RNA-seq data and tested it further by directly comparing the transcript structure predicted by the model to the transcription generated by the cell for synthetic DNA segments of random design. We show that the UM identifies transcription start sites more accurately than the initiation classifier alone, indicating that the relative arrangement of promoter and terminator elements influences their function. Our model presents a concrete description of how the cell defines transcript units, explains the existence of nongenic transcripts, and provides insight into genome evolution.

Identification of the cellular mechanisms that define gene structure has been a long-standing problem in molecular biology: It is of interest not only for the study of transcription and its regulation, but also for understanding how new genes arise (Carvunis et al. 2012), creating synthetic regulatory elements (Blount et al. 2012), and explaining the many types of “cryptic” transcripts (Xu et al. 2009; Yassour et al. 2010). While much is known about individual elements that function in eukaryotic transcription initiation and termination (see below), to our knowledge it has not been systematically explored which elements are critical on a genome-wide basis, what proportion of all transcription these elements can account for, and how they work together across entire chromosomes. For example, sequences that can function as promoters in some contexts may be disabled by transcription originating from an upstream or antisense promoter via repressive changes in the chromatin environment (Kaplan et al. 2003; Houseley et al. 2008; Bumgarner et al. 2009) or transcriptional interference (Mazo et al. 2007). Interactions in *cis* are also clearly important for transcriptional terminators, since a sequence can only be used to terminate a transcript if it is first transcribed. Moreover, the existence of physical connections between the 5′ and 3′ ends of genes that depend on proper 3′ end formation (Ansari and Hampsey 2005; Tan-Wong et al. 2012) suggests the existence of a feedback mechanism between terminators and promoters.

In eukaryotes, protein-coding genes are transcribed by RNA polymerase II (Pol II), which is loaded onto the promoter region via the general transcription factors (GTFs) (Juven-Gershon et al. 2008). However, the GTFs generally lack sequence specificity. In yeast, the only clear exception is the TATA-binding protein (TBP; encoded by the gene *SPT15*), which recognizes the TATA box. Although there appear to be TATA-like elements bound by Spt15 in most promoters (Rhee and Pugh 2012), only a minority have a canonical TATA box (Basehoar et al. 2004), and TATA boxes and TATA-like sequences also occur elsewhere in the genome. Motif matches for many yeast sequence-specific transcription factors (TFs) are enriched in promoters (Lee et al. 2007; Erb and van Nimwegen 2011), but it is not clear that they are sufficient to define transcription start sites (TSSs), as their activity is often dependent on other features of the promoter (Iyer and Struhl 1995; Sharon et al. 2012). Moreover, most yeast promoters have multiple TSSs, varying within a range of ∼26 bp (Pelechano et al. 2013). The exact start site appears to be controlled by the sequence surrounding the site (Chen and Struhl 1985; Hahn et al. 1985; Nagawa and Fink 1985) and has a consensus of YR (where Y = pyrimidine and R = purine) (Zhang and Dietrich 2005) which is likely recognized by TFIIB and/or Pol II (Pinto et al. 1992; Li et al. 1994; Bushnell et al. 2004).

In many eukaryotes, including yeast, a distinguishing feature of promoters is the presence of a nucleosome-depleted region (NDR) immediately upstream of the TSS (Yuan et al. 2005; Lee et al. 2007). Yeast promoters often contain a high A/T content and poly-dA:dT tracts, which inherently inhibit nucleosome formation (Iyer and Struhl 1995; Yuan et al. 2005; Kaplan et al. 2009; Tillo and Hughes 2009). Many promoters also contain binding sites for general regulatory factors (GRFs), including Rap1, Reb1, and Abf1 (Harbison et al. 2004), which cause nucleosome depletion in vivo where bound (Yu and Morse 1999; Yarragudi et al. 2004; Badis et al. 2008; Hartley and Madhani 2009; Kaplan et al. 2009; Ganapathi et al. 2011). However, none of these features are present in all promoters, and some occur outside of known promoters. Moreover, promoter definition is complicated by the fact that transcript initiation occurs bidirectionally in many promoter regions, with cryptic unstable transcripts (CUTs) produced by transcription in the antisense orientation (Neil et al. 2009; Xu et al. 2009). Promoters can further be regulated in *cis* by transcription over the promoter region, which results in transcriptional interference or histone modification (Kaplan et al. 2003; Martens et al. 2004; Mazo et al. 2007).

Termination of coding transcripts in yeast (and other eukaryotes) occurs when a cleavage and polyadenylation (CPA) site is recognized, mRNA cleavage occurs, and a polyadenine tail is added, producing a mature mRNA. Cleavage tends to occur over a range of about 36 bases and prefers to occur at (C/G)AA motifs (Pelechano et al. 2013), which may be recognized by the 3′ endonuclease Ysh1 (Mandel et al. 2006; Garas et al. 2008). Following cleavage, the nascent mRNA no longer has a 5′ m^7^G cap and becomes a substrate for 5′→3′ exonucleases that degrade the nascent RNA, leading to destabilization of the Pol II/RNA association and termination of transcription (Connelly and Manley 1988; Kim et al. 2004; West et al. 2004). CPA sites have been previously described as comprising several relatively simple motifs that are bound and recognized by the sequence-specific RNA-binding protein (RBP) components of the cleavage machinery. In yeast, these include the AU-rich efficiency element, bound by Hrp1 (Kessler et al. 1997; Chen and Hyman 1998), the A-rich positioning element, bound by Rna15 (Gross and Moore 2001), and several U-rich elements surrounding the cleavage site (likely bound by Yth1 and/or Cft1 [also known as Yhh1]) (Barabino et al. 1997; Dichtl et al. 2002; Tacahashi et al. 2003). In addition to mRNAs, Pol II synthesizes several types of noncoding transcripts, including snRNAs, snoRNAs, and CUTs, which have independent termination mechanisms (Jacquier 2009). For example, the RBPs Nab3 and Nrd1 recognize sequences present in RNAs and are important in the maturation of snoRNAs (Jacquier 2009) and the labeling of CUTs as TRAMP/exosome substrates (Wyers et al. 2005; Arigo et al. 2006). The CUT termination pathway provides a nonproductive termination mechanism for Pol II transcripts, as the resulting transcripts are immediately degraded.

Despite this extensive literature describing the sequences and factors involved, there has been no global examination of which features are critical to yeast gene identity, and therefore, the problem of how yeast (or any other eukaryote) delineates transcription units remains an open question. Many programs to identify genes in genomic DNA exist (e.g., Burge and Karlin 1997; Alexandersson et al. 2003; Solovyev and Shahmuradov 2003; Majoros et al. 2004), but these generally rely on sequence features unlikely to be used in the process of transcription, including sequence conservation and open reading frames (ORFs). Attempts to identify promoters (Prestridge 1995; Megraw et al. 2009) and CPA sites (Graber et al. 2002; Cheng et al. 2006) using mechanistic features, such as TF and RBP specificities, have met with varying success. To our knowledge, no study has taken an integrated approach to model the entire process of transcript definition or has tested the predictions of the model in vivo. Several groups have used synthetic promoter systems to assay the expression levels of pools of constructs (e.g., Gertz et al. 2009; Raveh-Sadka et al. 2012; Sharon et al. 2012). However, these studies aimed to measure how TF binding sites (TFBSs) or nucleosome positioning sequences affect expression level and so used a basal promoter sequence into which individual sequence elements were inserted or deleted. To our knowledge, no study has directly tested what sequences are necessary to generate a functional promoter in vivo.

Here, we have created a computer model that can explain genome-wide yeast transcript structure and is supported by multiple lines of experimental validation. The model is underpinned by classifiers that mimic the choices the cell makes when initiating and terminating transcripts. These classifiers indicate that the transcription of most yeast genes can be explained by relatively few features and reveal which *trans*-acting factors are most influential and which *cis*-elements help to define individual genes. Despite the fact that we did not incorporate CUTs into the training procedure, our model predicts bidirectional transcription from unidirectional promoters, indicating that the same sequence features generally drive transcript initiation for both mRNAs and CUTs and that CUTs are an inherent, and possibly unavoidable, feature of yeast promoters. Our model indicates that yeast CPA sites are also generally bidirectional, suggesting that convergent genes use the same termination elements. We combined these classifiers into a unified model that can predict where transcription will initiate more accurately than the initiation classifier alone, indicating that the relative arrangement of promoter and terminator elements in *cis* is a likely mechanism for orienting promoters. We go on to verify the predictions of the model using both existing data and experiments of our own design. This is the first model that describes how a eukaryotic cell defines transcript structure genome wide.

## Results

### Models of transcription initiation and termination

We first sought to ask how well we could explain the specific recognition of yeast promoters and CPA sites by cellular factors, using the known sequence features associated with these elements. This question can be framed as a computational classification problem, in which algorithms seek to classify input sequences as positives (e.g., promoter) or negatives (e.g., nonpromoter) on the basis of features in the sequences (e.g., TF motif scores at specific positions). We refer to the two resulting classifiers as the “initiation” and “termination” classifiers because they identify the regions where transcriptional units initiate (promoters) and terminate (CPA sites).

**Figure 1.**
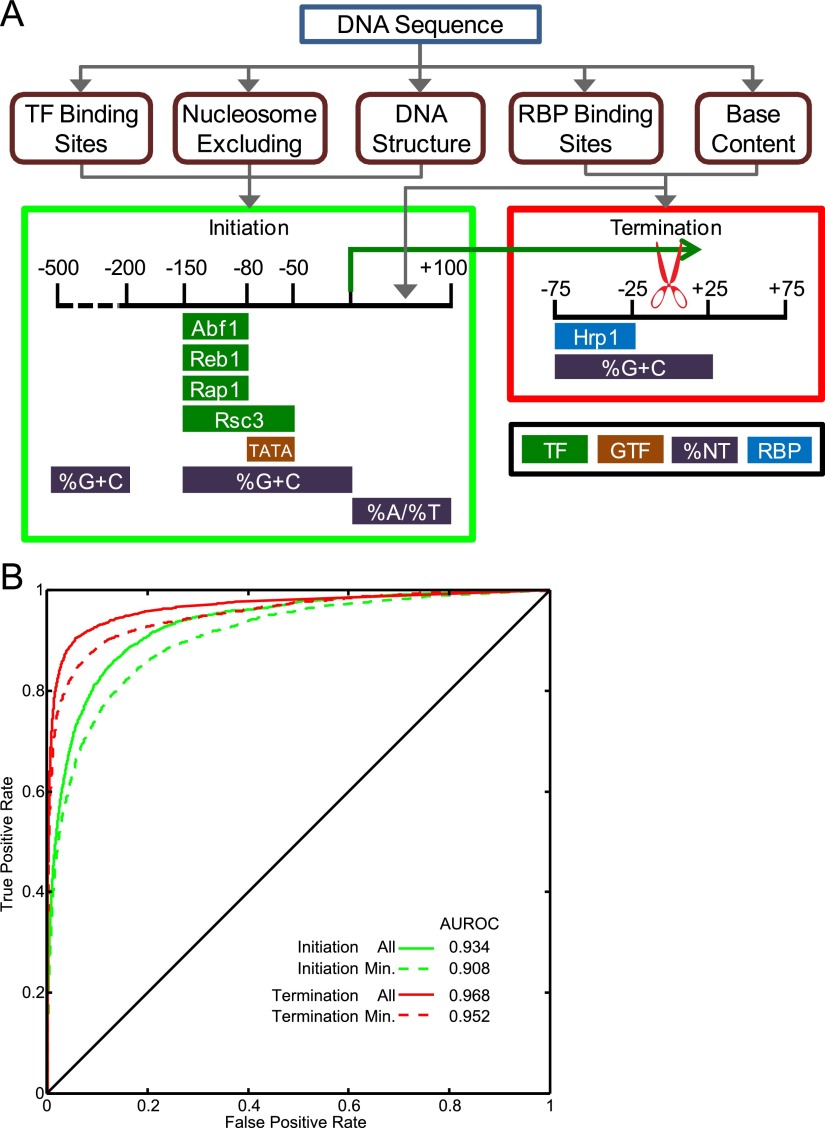
Design, refinement, and performance of the classifiers. (*A*) Classifier pipeline. Training and test examples were generated by calculating the relevant features (rounded boxes) using the DNA sequence of the example. The features were calculated over the bins shown in the colored boxes. At the *bottom* of the colored boxes, the components of the minimal feature sets are shown. Feature colors represent the feature type, including transcription factors (TF), general transcription factors (GTF), base content (%NT), or RNA-binding proteins (RBP). (*B*) ROC curves representing initiation and termination classifiers with either all features tested or the minimal feature sets, derived from the test data. The line *y* = *x* represents the curve expected by random classification.

Because we wanted the models to mimic cellular mechanisms, we restricted the input features to those that can be realistically sensed by nuclear factors, including predicted TF binding sites, nucleosome-excluding sequences, and DNA structural features, as well as the binding sites for RBPs and nucleotide content for genomic regions encompassing transcripts (since NTP concentrations can affect Pol II elongation rates) (Fig. 1A; Mason and Struhl 2005). For both RBPs and TFs, binding sites were predicted using motif models, rather than measured in vivo binding sites (i.e., ChIP data). We created an index of promoter and CPA sites of ORF-containing genes, using RNA-seq and tiling array data (see Supplemental Methods), which we used as positive examples. For the initiation classifier, we used nonpromoter sequences selected throughout the genome as negatives. For the termination classifier, we used sequences within transcripts as negatives (see Supplemental Methods). In order to calculate features, we subdivided these sequences into bins to reflect known or potential location preferences for specific features relative to TSS or CPA sites (see Supplemental Table 1; Supplemental Methods) and calculated a single score (e.g., a TF motif score) for each feature within each bin. This binning procedure limits the resolution of the classifiers, since they cannot identify the exact locations of features within bins, but it has two major advantages. First, it accounts for the fact that most yeast promoters and terminators use a range of initiation and cleavage sites (see Supplemental Fig. 1A,B; Pelechano et al. 2013). Second, it greatly reduces the number of features considered. A full list of the features initially included in each classifier is available in Supplemental Tables 2 and 3.

We created our classifiers using Random Forests (Breiman 2001), an approach that is capable of capturing nonlinear relationships between the features and classes, such as cooperative interactions among TFs (see Supplemental Methods). To produce positive examples for the two classifiers, we first identified TSSs and CPA sites for protein-coding genes. We defined TSSs using those observed in Lipson et al. (2009) and CPA sites using RNA-seq reads containing poly(A) sequences (see Supplemental Methods; Nagalakshmi et al. 2008). This yielded 5010 genes with both TSS and CPA sites. We scored the initiation classifier features in six bins, as shown in Figure 1A, for 600 bp surrounding the TSS (from −500 to +100, relative to the TSS). We obtained negative examples by dividing nonpromoter portions of the genome into overlapping 600-bp windows, yielding 72,276 negative examples. For the termination classifier, we scored features in three 50-base bins encompassing 150 bases surrounding the CPA site (from −75 to +75, relative to the CPA site) (see Fig. 1A). We derived negative examples by dividing sense ORFs into overlapping 150-bp segments, yielding 155,093 negative examples. A complete description of the sequence selection rules is given in the Supplemental Methods. We split the genome into two halves (chromosomes 1–8 and 9–16), using half to train the classifiers and leaving the other half of the genome for testing the model. Within each training half, we created eight random forests, each training on seven chromosomes and withholding one chromosome for model refinement. The predictions from the eight forests were then averaged to produce a classification score for the test data. Figure 1B (and Supplemental Fig. 2) shows the performance of the classifiers in segregating the positives from negatives in the test data.

To minimize overfitting of the models, remove redundant features, and make the models easier to interpret, we sought to reduce the number of included features. We did this by iteratively rebuilding the model, adding one feature at a time, with features sorted by decreasing “importance” (as defined by the random forests algorithm), and retaining only those that appreciably improved the performance of the model measured on the “refinement” data (see Supplemental Methods). This feature reduction procedure only slightly decreased the performance of each classifier on the “test” data, while greatly reducing the numbers of features (Fig. 1B; Supplemental Fig. 2). A striking result of this procedure is that the two classifiers are dominated by a small number of critical feature types: We retained a total of 15 features for the initiation classifier and four features for the termination classifier (Fig. 1A; Supplemental Fig. 3). We also found that, using these reduced feature sets, a linear model (logistic regression) performed only marginally worse than the Random Forests model (AUROC of 0.901 vs. 0.908 for initiation, and 0.946 vs. 0.952 for termination), indicating that there are few important nonlinear feature interactions. Indeed, the important *trans*-acting factors tend to bind distinct sets of promoters (Supplemental Fig. 3E), consistent with independent function.

The initiation classifier was largely dependent (14/15 features) on six main types of features: binding sites for the GRFs Reb1, Abf1, and Rap1; binding sites for Rsc3 (a putative GRF and component of the RSC complex) (Badis et al. 2008); DNA structural features and poly(A) tracts (which correlate with G/C content and presumably serve to deplete nucleosomes, and so are hereafter described collectively as “G/C content” features); and TATA boxes (Fig. 1A). A classifier with this reduced feature set has an AUROC only 2.6% lower than one containing all 1698 features (AUROC = 0.908 vs. 0.934) (Fig. 1B), suggesting that these signals are responsible for establishing the identity of the vast majority of yeast promoters.

**Figure 2.**
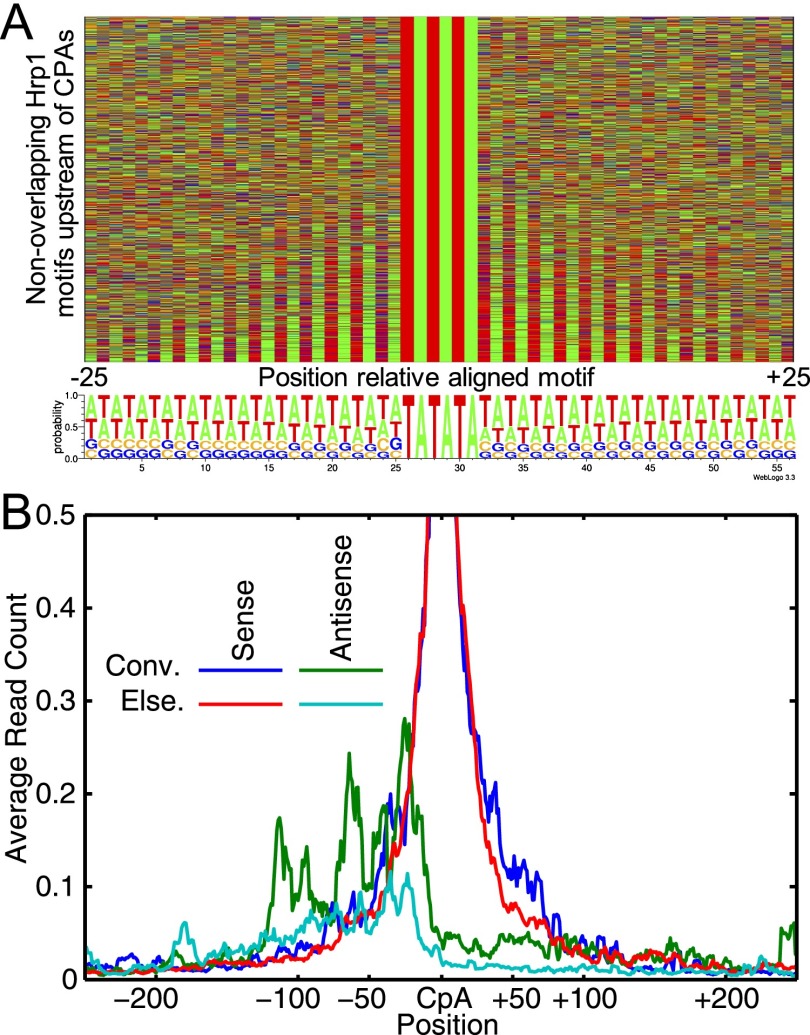
Properties of terminators. (*A*) Base content surrounding the 3457 optimal nonoverlapping Hrp1 binding sites in terminator regions (≤150 bp upstream of CPA site). Colors indicate the base at the corresponding position, from 25 bp upstream of to 25 bp downstream from the motif match. (*B*) Alignment of RNA-seq reads corresponding to poly(A) sites (Nagalakshmi et al. 2008) on both DNA strands for convergent intergenic regions (Conv.) and elsewhere in the genome (Else.). Data are aligned to sense poly(A) sites and include all poly(A) sites in the genome with at least two reads. The data represent the average read count of all aligned loci and are smoothed over a 5-bp window.

The termination classifier was reduced to only two feature types (Fig. 1A): G/C content and Hrp1 binding sites. Together, these confer an AUROC of 0.952 on the test data, only slightly lower than that obtained when all 147 features are included (AUROC = 0.968). We initially included A- and U-rich motifs to capture the canonical sequence specificities of the other CPA factors (Barabino et al. 1997; Gross and Moore 2001; Dichtl et al. 2002; Tacahashi et al. 2003), but these were not selected by the above procedure. This result might be explained by several possibilities: The sequence preferences of these factors may be better captured by base content than by the motifs; the motifs may not accurately capture the specificities of the factors; or the factors may not strongly impact cleavage site selection. G/C content can also impact nucleosome occupancy, which appears low at terminators, although the significance of this observation is unknown (Chung et al. 2010; Fan et al. 2010; Allan et al. 2012; Brogaard et al. 2012). Strikingly, Hrp1 binding sites in terminator regions are often flanked by additional TA repeats that tend to remain in phase (Fig. 2A). Hrp1 can occupy two directly adjacent TA(3) motifs (Perez-Canadillas 2006). However, the overall pattern of Hrp1 motif occurrence in terminator regions is inconsistent with cooperative binding (Supplemental Methods), so this pattern may simply reflect the fact that each additional TA di-nucleotide creates another potential Hrp1 binding site.

We next asked how well the classifiers identify the TSS and CPA sites on a per-base level. Despite the low intrinsic resolution of the predictors (∼70 bp for the initiation classifier and ∼50 bp for the termination classifier, due to the bin sizes), the majority of bases used as TSSs/CPA sites in promoter/terminator regions (Pelechano et al. 2013) lie near the center of these peaks (Supplemental Fig. 1C,D). Moreover, searching for the initiator (CA) and the cleavage site ([G/C]AA) motifs within the peaks often identifies the exact TSS or CPA site (Supplemental Fig. 1E,F; see Supplemental Methods), consistent with earlier observations that the exact transcript start and end bases are determined by local sequence cues, following identification of the general regions for transcript initiation (Hahn et al. 1985; Nagawa and Fink 1985) and termination (Russo et al. 1991).

### Both promoters and CPA sites are bidirectional

Strikingly, the key features for both the classifiers contain little strand specificity. The most important features of the initiation classifier (GRF sites and G/C content/poly[A] sequences) are thought to function through their role in NDR formation (Iyer and Struhl 1995; Yu and Morse 1999; Lee et al. 2007; Badis et al. 2008; Kaplan et al. 2009; Tillo and Hughes 2009) and so should function bidirectionally, since NDRs have no orientation. Indeed, for the initiation classifier, the predictions for the forward and reverse strands in the test set are highly correlated (Pearson R ≈ 0.5), and the correlation is maximal when the DNA strands are offset by −208 bp, relative to the forward strand. Thus, the initiation classifier predicts that many promoters initiate transcripts in either orientation. Indeed, the average distance between the predicted sense and antisense TSSs (212 bp) is consistent with bidirectional initiation observed in vivo (Neil et al. 2009).

Both the Hrp1 motif and G/C content are symmetric, and correspondingly, the termination classifier's predictions are also highly correlated between the forward and reverse DNA strands (Pearson R ≈ 0.8 when offset by 90 bp), suggesting that the same cleavage signals can operate in either orientation. In fact, 42% of convergent genes have only one optimal Hrp1 site between them. Further, RNA-seq reads containing poly(A) sequences (Nagalakshmi et al. 2008) indicate that a substantial fraction of CPA sites terminate transcripts in both orientations, that this is especially common for CPA sites between convergent genes, and that the distance between cleavage sites on either strand is consistent with the same cleavage signals being used to terminate both transcripts (Fig. 2B).

### Promoter-defining elements drive transcription in vivo

To our knowledge, our classifiers represent the first rigorous demonstration that the few features highlighted can account for the identity of most yeast gene structures. We next sought experimental evidence that the features are necessary and sufficient in vivo. We began by examining published expression data sets corresponding to mutations in the key *trans*-acting factors: Abf1, Reb1, Rap1, Rsc3, and Spt15 (TBP) (Alper et al. 2006; Badis et al. 2008). For a given DNA sequence, our model provides a score for how “promoter-like” a sequence is (from 0 to 1). We considered a gene's promoter to depend on a factor if leaving out the corresponding feature reduced the promoter's score by at least 0.1. In general, the expression of genes whose promoters are predicted to depend on a factor changes significantly more in the corresponding mutant (by rank sum test) (see Supplemental Methods; Supplemental Fig. 4). This finding demonstrates that the initiation classifier can identify the set of genes controlled by the predicted promoter-defining factors. Consistent with the notion that there is little interaction between promoter-defining features, motif scores for the individual TFs perturbed in each experiment predict the genes affected nearly as well as the initiation classifier (Supplemental Fig. 4).

**Figure 3.**
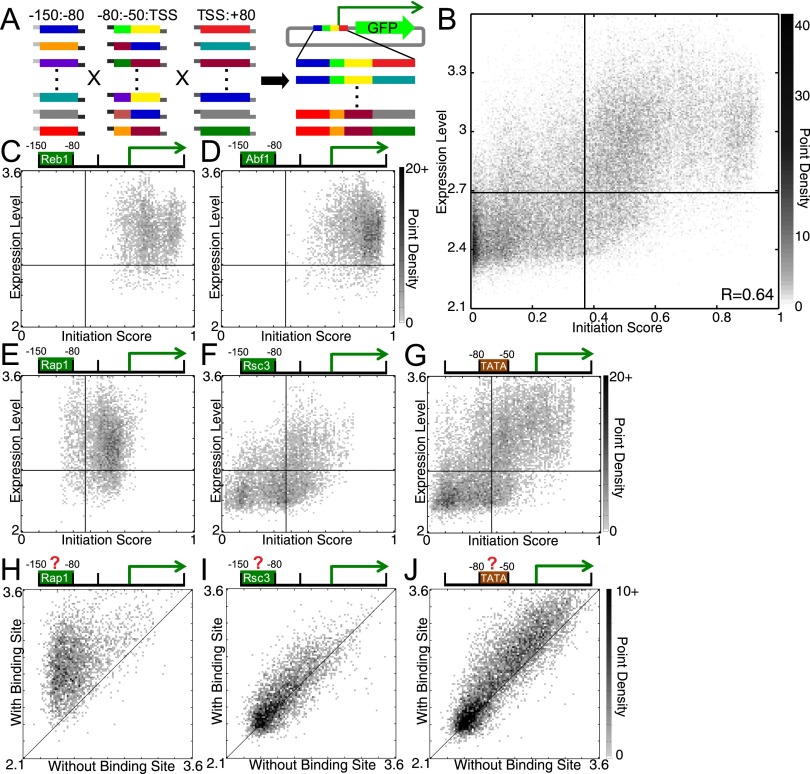
Construction and analysis of the combinatorial promoter library. (*A*) Synthetic double-stranded promoter fragments with complementary overhangs were ligated together to yield full-length promoters, which were then cloned into a GFP expression vector. We used flow cytometry and sequencing to measure the expression level of each promoter (see Methods). (*B*) Point-density scatter plot showing the correlation between the initiation score and the expression level (as described in Methods, log-scale). Darkness corresponds to point density. Horizontal and vertical lines indicate the expression level and initiation score thresholds for considering sequences “expressed” and a “predicted promoter,” respectively. (*C–G*) Identical to *B* but divided into promoters containing (*C*) Reb1, (*D*) Abf1, (*E*) Rap1, and (*F*) Rsc3 binding sites in the −150:−80 bin, and (*G*) the TATA box in the −80:−50 bin. (*H–J*) Point-density scatter plots showing the expression level of promoters that are identical except for the presence or absence of functional (*H*) Rap1, (*I*) Rsc3, or (*J*) Spt15 (TBP) binding sites. The line *y* = *x* marks the point at which expression is identical between the two promoters, regardless of the binding site's presence. The other GRFs (Abf1 and Reb1) are similar to Rap1 (*H*).

We next tested whether these *cis*-elements are sufficient to initiate gene expression in vivo using a combinatorial library of promoter constructs driving GFP, embedded in a context that otherwise has no promoter-like properties. These constructs encompassed the parts of the promoter most critical to the model (from −150 to +80 relative to the TSS). We designed the sequences in three different sections (Fig. 3A) encompassing four of the bins used in the model (one of the sections encompassed two bins). The designed sequences were selected computationally from a large excess of randomly generated sequences of varying G/C content, with or without sites for Abf1, Reb1, Rap1, Rsc3, or Spt15 randomly placed within the regions in which they are relevant to the model. Some of the sequences are predicted to form functional promoters by virtue of their G/C content alone. For each fragment containing a TFBS, we synthesized a fragment that is identical except that the TFBS was disrupted at several key bases. We used conventional oligonucleotide synthesis followed by pooled ligations to create a library of promoters from these promoter fragments that theoretically contained 86,688 distinct sequences. We assayed the relative activity of each promoter in yeast by using cell sorting and sequencing to estimate the expression level of each promoter, similar to a procedure used previously (see Methods and Supplemental Methods for details; Sharon et al. 2012), yielding sufficient data to estimate the expression level of 48,928 different promoters.

We obtained a strong and significant correlation between initiation score (a number between 0 and 1) and GFP expression level (Spearman R = 0.64, i.e., 41% [R^2^] of variance in ranks explained *P* ≈ 0) (Fig. 3B), indicating that the features of the promoter model are sufficient for promoter function. At an optimal threshold (promoter score = 0.37, GFP expression level = 2.69), the promoter classifier achieves a true positive rate of 73%, with a false positive rate of only 21%. Promoters containing Rap1, Reb1, and Abf1 binding sites, to which the model assigns high scores, are nearly always expressed (expression > 2.69) (Fig. 3C–E), and the expression level is consistently higher than promoters that are identical except for disruption of the binding site (e.g., Fig. 3H). The impact of Rsc3 sites and the TATA box appear weaker; many promoters containing these elements are neither expressed nor predicted to be functional promoters (Fig. 3F,G). Nonetheless, comparison of GFP levels from the promoters that are identical except for the presence and absence of Rsc3 or TATA elements showed that these elements have a positive impact on promoter function (Fig. 3I,J). Overall, the model explains a considerable amount of the variation in GFP expression; the additional diversity in expression may be due to experimental variability as well as the presence of TFBSs that affect gene expression level, but not promoter identity, and so were not considered in our analysis.

### A unified model improves computational gene identification

**Figure 4.**
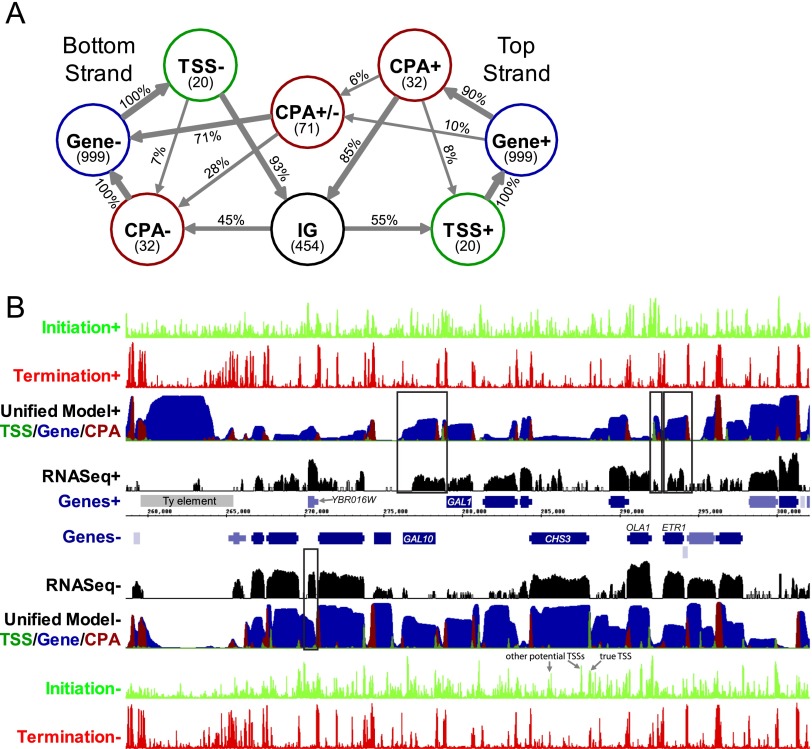
A genome-scale yeast transcript model. (*A*) The structure of the Unified Model HMM. Circles represent states and arrows represent interstate transitions. Inside state circles, the number of bases the model expects to remain in each state is shown in parentheses. Transition probabilities, as a percent of outgoing transitions, are shown on transition arrows. Very infrequent transitions (probability < 1%) are not shown. (IG) Intergenic state. (*B*) Genome Browser display illustrating the predictions of the models at the *GAL1-10* locus of chromosome 2. The tracks on the *top* half represent data for the forward strand of DNA, with the reverse strand on the lower half. From center: blue bars represent genes, with thinner bars representing UTRs, and the gray bar represents a Ty element. Black tracks represent RNA-seq read density on a log scale (Levin et al. 2010). The Unified Model's predictions are shown with dark green, blue, and red on a single track representing the probability of being in each of the states, where the probabilities are shown stacked. The light green and red tracks on the outer edge represent the scores for the initiation and termination classifiers, respectively. Initiation peaks corresponding to the true TSS and other potential TSSs for the *CHS3* gene are as indicated, and some examples of predicted nongenic transcripts that are supported by RNA-seq are shown boxed.

We next created a “Unified Model” (UM) aimed at describing the full process of transcription. The UM uses a hidden Markov model (HMM), which is ideal for this purpose because it can probabilistically model the states of Pol II as it initiates, transcribes, and terminates transcription in a similar way to how it occurs in the cell. In essence, an HMM takes as inputs a model structure (as in Fig. 4A) and a set of “observations” (i.e., the classifier scores for each base of a chromosome) and outputs the probability of the model being in each state at each base (i.e., chromosome-wide transcript structures). The model structure includes parameters describing the probability distributions of the observations in each state (i.e., the distributions of the classifier scores within each of the states shown in Fig. 4A), as well as the probability distribution of transitions between states (e.g., the probability of going from intergenic to TSS+). Since there are eight different states and four different observations (two classifiers for both DNA strands) (Fig. 4A), the model requires 32 (8 × 4) means and 32 (8 × 4) variances for observations, as well as 64 (8 × 8) different transition probabilities (40 of which are zero, since not all state transitions are allowed, e.g., Gene+ directly to Gene−). To ensure that the model is symmetric, such that the predicted transcript structure will be identical for a chromosome sequence and its reverse complement (since a chromosome's strand labels are arbitrarily assigned), we used the same means and variances for equivalent states between the (+) and (−) strands and ensured that the transition probabilities were symmetrical, resulting in a total of 56 nonzero parameters (see Supplemental Tables 4, 5).

The UM states correspond to TSSs, transcript bodies (“Gene”), and CPA sites on the forward and reverse strands, as well as a state corresponding to bidirectional transcript termination and a state for intergenic DNA (Fig. 4A). The model considers transcription in both orientations simultaneously, scanning in only one direction. The order of the states for transcripts on the reverse strand is thus reversed relative to the forward strand, e.g., while the TSS is followed by a gene on the forward strand, the TSS is followed by an intergenic region on the reverse strand. Because TSS and CPA sites typically cover a range of bases (Pelechano et al. 2013), we represented TSS and CPA sites as states that can span multiple bases. As a result, we could not use our original transcript map (the one used to train the initiation and termination classifiers) to derive the UM parameters because it has only a single base for each TSS and CPA site and so does not capture the actual range of bases used for these sites or the transitions between these and the other model states. Further, these annotations excluded many cellular RNAs, including ORF-containing genes for which we did not have TSS/CPA site estimates.

To overcome these limitations, we defined a new transcript map that could be used in deriving the HMM parameters. In our initial analyses (data not shown), the model outputs (i.e., the predicted transcript structures) were surprisingly sensitive to the choice of parameters and the transcript maps from which they were derived. Ultimately, we derived the parameters using a transcript map that divided the genome into the eight HMM states based on multiple types of RNA-seq data (Nagalakshmi et al. 2008; Lipson et al. 2009; Levin et al. 2010; Ozsolak et al. 2010) (see Supplemental Methods and Supplemental Fig. 5 for details). We also found that the performance of the UM could be substantially improved by tuning the observation means (i.e., the expected classifier score in each state) to maximize the correlation of the probability of being a transcript (as predicted by the UM) to the expression levels in the training data, measured by RNA-seq on a per-base level (see Supplemental Methods for details; Levin et al. 2010). We speculate that this process may compensate for errors or omissions in the initial transcript map. It is also possible that some aspects of transcription are not captured well by the binary state annotations in our transcript map. Here we present results for the UM trained on chromosomes 9–16 and tested on chromosomes 1–8 using scores from initiation and termination classifiers trained on chromosomes 9–16 (ensuring neither UM nor classifiers had seen these chromosomes previously). Similar model parameters and performance were attained by swapping the training and test data.

**Figure 5.**
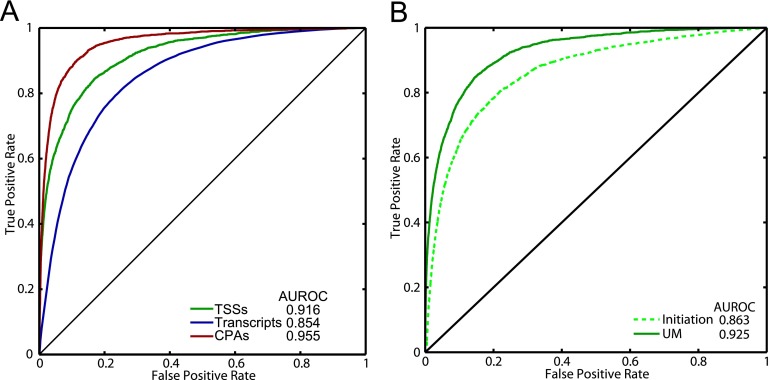
Performance of the UM. (*A*) ROC curves illustrating how well the UM predicts TSSs, transcripts, and CPA sites, when classifying the positive and negative examples for the initiation and termination classifiers, as well as ORFs/transcripts and nontranscript bases. (*B*) ROC curve comparing the ability of both the UM and initiation classifier to distinguish between TSSs and bases that are part of nondubious ORFs. The line *y* = *x* represents the curve expected by random classification.

Given the classifier scores for an entire chromosome, the UM outputs the probability of being in each state at every base. The probability corresponds to the posterior marginals of the HMM states. An example of our UM predictions at the *GAL1-10* locus (and surrounding region), located on chromosome 2, is shown in Figure 4B. There is generally a good correspondence between gene annotations and the transcript predictions (and the exceptions are informative; see below). The UM robustly predicts TSSs, CPAs, and transcripts of known genes: Figure 5A shows ROC analysis, and Supplemental Figure 6 shows the same data as precision-recall curves. The overall sensitivity (recall) of detecting transcripts on a base-by-base level is 76.6% (*P* ≈ 0), and the precision is 76.9% (*P* < 10^−299^, taken as predicted transcribed bases that are within 100 bp of a known transcript) (see Supplemental Methods). For comparison, random guessing with a probability of 42.1% (the proportion of the genome encompassed by a transcript on a given strand) would yield precision and recall values of 42.1%. TSSs and CPAs are much more sparse than transcripts, yet the ROC and precision-recall analyses indicate that the UM is also adept at identifying these elements (Fig. 5A; Supplemental Fig. 6). We did not expect the UM to achieve perfect classification in this analysis because it is penalized for predicting any transcript that is absent from our gene annotations, even if these transcripts exist in the cell (see below). Further, if the UM predicts an antisense over a sense transcript, it is penalized twice in this analysis: Predicting the antisense transcript yields false positives, and missing the sense transcript yields false negatives.

The UM also allowed us to ask whether the interaction of sequence features along the chromosome is important to their function in vivo. If so, the UM would be expected to be better at identifying TSSs than the initiation classifier alone. Indeed, the UM is much better than the initiation classifier at distinguishing true TSSs from bases that are within ORFs (AUROC = 0.925 vs. 0.863, representing nearly a halving of the error rate) (Fig. 5B), indicating that the relative arrangement of promoter and CPA sites on the chromosome has an important influence on the usage of the individual elements and that this context-dependence is being modeled by the UM. Manual examination of the UM and the classifier outputs confirm that the HMM often identifies the correct TSS for a given gene even when the initiation classifier identifies other, often stronger, potential TSSs nearby, suggesting that this context-dependence is being incorporated in the UM. *CHS3* in Figure 4B is one such example (indicated with arrows).

We next investigated the nongenic transcripts predicted by the model. Transposons and sn/snoRNAs, although not considered as known transcripts in the analyses above, are also generated by Pol II (Lesage and Todeschini 2005; Richard and Manley 2009). These elements encompass 2.95% of predicted transcribed intergenic bases, and overall 71.3% of the bases of these elements are predicted to be transcribed, despite these elements being absent from the classifier training data (*P* < 10^−7^). Remaining inconsistencies between the model and the annotated transcripts are split approximately evenly between predicting transcription on the wrong strand (15.3% of predicted transcribed bases) and predicting transcripts in intergenic regions (>100 bases from ORF-containing transcripts; 11.7% of predicted transcribed bases). Figure 4B contains several such instances. In addition to capturing most of the known genes, the model identifies a known antisense transcript of *GAL10* (Houseley et al. 2008), transcripts antisense to *ETR1* and *YBR016W*, and a short intergenic transcript between *ETR1* and *OLA1*. Notably, all are also observed in the RNA-seq data.

In order to determine more globally whether there was evidence to support the existence of transcripts that do not correspond to known features, we compared the UM's predictions to available RNA-seq data. Among the transcripts predicted antisense to known genes, 23.5% of bases are supported by strand-specific RNA-seq reads (Lipson et al. 2009) (compared with 8.5% expected by chance; *P* ≈ 0), and 39.0% of intergenic bases that are predicted to be transcribed are supported by the same RNA-seq data (compared with 25.6% expected by chance; *P* ≈ 0). Finally, examining complete transcript predictions that do not overlap known features (see Supplemental Methods), we find that available RNA-seq (Levin et al. 2010) and NET-seq (Churchman and Weissman 2011) data show that the predicted TSSs and CPA sites of nongenic transcripts appear in aggregate to be largely correct (Supplemental Fig. 7). These comparisons demonstrate that the model is capturing many real nongenic transcripts and suggest that these transcripts are produced by the same mechanisms that produce conventional Pol II transcripts.

### Transcripts produced from randomly generated DNA sequences

An intriguing feature of our classifiers and the UM is that the critical sequence features are relatively simple and would therefore be expected to arise in random sequence at a relatively high frequency. For example, the binding sites for Reb1, Abf1, and Rap1 have only 7, 7, and 9 critical bases, respectively, and tolerate some degeneracy; thus, binding sites for at least one of these factors should appear approximately every 2 kb in a randomly generated sequence. Hrp1 binding sites, with a six-base optimal binding site, appear even more frequently—roughly once every kb in the A/T-rich yeast genome. Thus, relatively long transcripts should be produced with some frequency even from a randomly generated DNA sequence.

To confirm that such transcripts do arise and are consistent with the predictions of the UM, we assayed transcription in vivo from synthetic DNA fragments that were integrated into the yeast genome. We tested four 6-kb fragments, each composed of two tandem 3-kb DNA fragments, denoted A1B1, A1B2, A2B1, and A2B2. These fragments contained randomly generated sequences with a G/C content similar to that of the yeast genome (38%), into which we randomly inserted binding sites for Rap1, Abf1, Rsc3, Reb1, and Spt15, to reduce the amount of DNA to be synthesized (see Supplemental Methods). The 3-kb fragments we synthesized contained an average of 3.75 Spt15, 2.25 Abf1, 0.75 Reb1, 0.25 Rap1, and 3.25 Rsc3 consensus binding sites each, which is only slightly more than the number expected in completely random sequences of the same length (2.56, 0.99, 0.24, 0.24, and 1.2 binding sites, respectively). We did not intentionally add CPA sites because they appear very frequently in randomly generated DNA sequences. We integrated these constructs into the genome and assayed expression using a custom tiling array.

**Figure 6.**
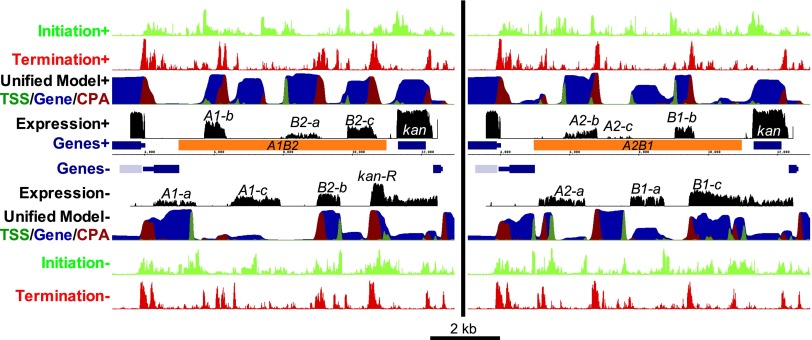
Predicted transcript structure and measured expression of two of the four randomly generated 6-kb fragments. Tracks as in Figure 4, except that “Expression” was measured using custom Agilent tiling arrays. The construct names are indicated in the *center*, and discernible transcripts are labeled along the tiling array data. A1B1 and A2B2 constructs are shown in Supplemental Figure 8 and show very similar results.

The expression data shown in Figure 6 and Supplemental Figure 8 illustrate that all four fragments produce a diverse set of transcripts whose expression levels span three orders of magnitude. The UM predicts the majority of transcript species correctly. There are several instances of promoters that initiate transcripts bidirectionally (*A1-a*/*A1-b*, *B1-a*/*B1-b*, *B2-b*/*B2-c*), as well as numerous instances of convergent transcripts that appear to terminate at the same bidirectional CPA site (*B1-b*/*B1-c*, *B2-a*/*B2-b*, *A2-c*/*B1-a, B2-c*/*kan-R*). Of the 12 easily distinguished transcripts, nine are robustly predicted by the UM (*A1-a*, *A1-b*, *A2-b*, *B1-a*, *B1-b*, *B2-a*, *B2-b*, *B2-c*, *kan-R*). There are several examples where transcripts are generated on both DNA strands. These cases are difficult for the UM to predict because it has no states representing transcripts on both strands; nonetheless, in both instances of overlapping transcripts (*A2-a*/*A2-b* and *kan* and transcripts generated antisense to *kan*), the model is uncertain and partially predicts transcripts on both strands. In other cases, it is uncertain which strand is transcribed when the data support the transcription of only one strand under the condition tested (*A1-c*, *B1-a*, *B2-c*). Of the easily distinguished transcripts, our model achieves a precision and a recall of 0.69, on a transcript-by-transcript basis, allowing a 200-base offset for TSS and CPA sites. Because the model is incapable of simultaneously predicting both of the overlapping transcripts *A2-a* and *A2-b*, the theoretical maximum is a precision and recall of 0.92. By simulating random guessing for the positions and strands of transcripts, but assuming the number and sizes of transcripts are known, the upper bound of what is expected by chance is a precision and recall of about 0.029. Altogether, these results demonstrate that transcripts can arise from a pseudorandom sequence at a predictable frequency.

## Discussion

The modeling and experimental validation presented here indicate that the majority of mRNA transcript definition in *Saccharomyces cerevisiae* is relatively simple, and that transcription occurs as a probabilistic process that can be faithfully captured in a hidden Markov model. Both the models and the experimental data described here indicate that, rather than simply being a property of promoters, promoters are defined by the presence of an NDR, resulting from sequences that inherently inhibit nucleosome formation, such as G/C content and poly(A) sequences, or binding sites for a small number of chromatin-modifying TFs, mainly GRFs. Most of the features originally tested in the initiation classifier correspond to motifs for other TFs, and so the slight loss in the initiation classifier's performance when reducing the number of features (Fig. 1B) may be explained by less frequent utilization of other mechanisms, such as the ability of arrays of TFBSs to exclude nucleosomes and promote transcription (Pettersson and Schaffner 1990; Adams and Workman 1995; Miller and Widom 2003; Bai et al. 2011). However, the fact that a linear model performed as well as Random Forests suggests that the major promoter-defining elements do not work cooperatively.

**Figure 7.**
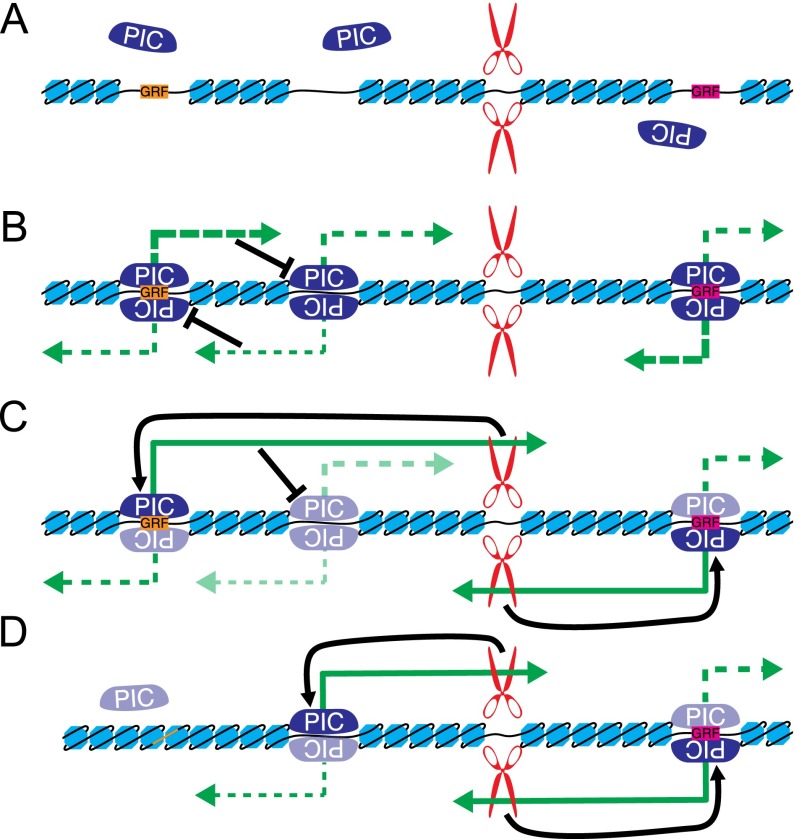
Gene definition model. (*A*) In the absence of transcription, the DNA forms nucleosomes except where prevented by bound TFs (such as the GRFs) or by the DNA structure. (*B*) Transcription begins indiscriminately from nucleosome-free regions in proportion to the efficiency of pre-initiation complex (PIC) formation. Promoters compete with one another in *cis* through the act of transcription. (*C*) An equilibrium is reached where some promoters are active and others are repressed. Successful cleavage and polyadenylation reinforces the promoter choice. (*D*) If a nucleosome-free region is destroyed (for instance, through loss of GRF binding), it is no longer competent for initiating transcription. Downstream promoters are then de-repressed, become active, and a new equilibrium is reached.

Importantly, our model captures the interactions between these features in *cis*: The fact that the UM is more accurate at identifying promoters than the initiation classifier alone indicates that promoter selection can be influenced by cleavage site usage and the relative arrangement of these elements on the chromosome (Fig. 5B). Although previous studies have characterized potential mechanisms for these types of *cis* interactions in the regulation of specific genes, our model suggests that they are of widespread importance. One possible mechanism for this influence is through gene looping, which is a physical connection between the 5′ and 3′ ends of genes dependent on proper 3′ end formation (Ansari and Hampsey 2005; Tan-Wong et al. 2012). Following pioneering rounds of transcription from all promoter-like sequences, successful CPA events may reinforce the “correct” transcript choice (Fig. 7). Previous studies have also established that transcription can influence the function of nearby elements by transcriptional interference (Mazo et al. 2007) and repressive changes in the chromatin environment of the downstream promoter (Kaplan et al. 2003). These mechanisms would force promoters to compete with one another in *cis*, with factors such as initiation frequency, epigenetic state, and gene loop formation determining which promoter becomes dominant (Fig. 7). Given this model, we would expect that under conditions where an upstream promoter is inactivated, downstream promoters could become active as repressive transcription from the upstream promoter stops. Indeed, we can find many potential examples of this phenomenon (Supplemental Fig. 9). Thus, while few TFBSs contributed to the initiation classifier, they could help determine which among competing promoters is dominant, since the relative activation levels of promoters near each other can influence which transcripts are produced in the neighborhood. Such a mechanism may also explain why it is beneficial to incorporate expression levels in training the UM parameters: Their inclusion indirectly informs the model about the global transcriptional state of the cell.

The initiation classifier explains the presence of many noncoding transcripts. Previously reported cryptic transcripts, including CUTs, SUTs, and antisense transcripts (Xu et al. 2009; Yassour et al. 2010), are all, on average, associated with promoter-like sequences at the 5′ end (Supplemental Fig. 10). Many of these transcripts initiate from bidirectional promoters (Neil et al. 2009; Xu et al. 2009). In our analysis, the correlation between the initiation classifier's predictions for the forward and reverse DNA strands is only about 0.5, suggesting that there is some asymmetry incorporated into the promoter itself; indeed, the distribution of the critical promoter-defining features is only partly symmetric (Fig. 1A). It is possible that either the arrangement or orientation of binding sites for some of the other ∼200 TFs in *S. cerevisiae* help to make initiation more efficient in one direction. The base content of the nascent transcript could also be a contributor to asymmetrical transcript initiation from bidirectional promoters. We found that the ratio of As to Ts is important in the first 100 transcribed bases, which could help to control the efficiency of Pol II promoter release, since elongation rate can be affected by nucleotide content (Mason and Struhl 2005).

The termination classifier is both remarkably accurate (Fig. 1B) and very simple, depending on only base content and Hrp1 sites. Historically, the Hrp1 binding site was thought to control the efficiency of downstream cleavage, while selection of the site of cleavage was determined by the positioning element (Guo and Sherman 1996). Our model is partly consistent with this view and further suggests that the general location of cleavage is determined primarily by Hrp1-binding, with a minor but significant contribution by A/T-content to cleavage site identity. The palindromic nature of the features favored by the termination classifier strongly supports the bidirectional nature of yeast cleavage sites. It has previously been shown that some yeast terminators can stimulate cleavage and polyadenylation in either orientation (Peterson and Myers 1993; Egli et al. 1997; Aranda et al. 1998) and that the 3′ ends of convergent yeast genes frequently overlap (Nagalakshmi et al. 2008; Ozsolak et al. 2010). We provide evidence that the same *cis* elements are generally used to stimulate transcript termination in either orientation. In fact, our UM predicts that over 40% of convergent genes have overlapping terminator regions (see Supplemental Methods). Such a mechanism could have a role in preventing transcriptional interference between adjacent genes by minimizing overlapping transcription and could also contribute to genome compaction.

The simplicity of our model means that gene structures can easily be designed and should arise spontaneously in evolution. Yeast is a prevalent system in synthetic biology (e.g., Khalil et al. 2012; Wei et al. 2012; Westfall et al. 2012). However, most of the promoter and terminator sequences currently in use are based on native sequences and so have the potential to recombine (Blount et al. 2012). Our model provides a guide with which these elements can be designed de novo. The simplicity of these elements also means that they can easily arise over evolutionary time. For promoters, all that is fundamentally required is the gradual expansion of A/T-rich tracts and/or addition of binding sites for the five TFs that dominate the model. Indeed, promoter-like sequences will occur in randomly generated DNA sequences at a rate of ∼1/kb (see Supplemental Methods). Since a given promoter-sized sequence is likely to contain multiple TFBSs purely by chance (there are, on average, 27 perfect TFBSs per kilobase of random DNA drawn from the yeast base composition, using the YeTFaSCo expert-curated motifs [de Boer and Hughes 2012]), then a newly emerged promoter will likely already be regulated in some way. The fact that functional variants can arise frequently at random could explain both the relatively high evolutionary rates in *cis*-regulatory sequence (Kellis et al. 2003; Weirauch and Hughes 2010) and de novo gene birth. Creating new genes from essentially random DNA was thought to be very unlikely (Jacob 1977), but several recent studies have shown that it may be more common than previously thought (Levine et al. 2006; Cai et al. 2008; Carvunis et al. 2012). Consistent with this, our UM predicts that 66% of bases in randomly generated DNA sequences will be transcribed on one strand or another. One model for the origin of new genes involves first generating a stable transcript, then acquiring coding potential, and finally acquiring a function (Cai et al. 2008). However, until now, it was unclear how simple it is to generate stable transcriptional units from essentially random intergenic DNA.

## Methods

Supporting data and model predictions are available on our website (http://hugheslab.ccbr.utoronto.ca/supplementary-data/transcription_model/). Additional methodological descriptions are available in the Supplemental Methods and Analysis.

### Creating the initiation and termination classifiers

For the initiation classifier, features included the YeTFaSCo database's Expert-Curated set of TF motifs (version 1.00) (de Boer and Hughes 2012), nucleosome-excluding sequences, and DNA structural features (Supplemental Table 2). The termination classifier used a set of RBP motifs, as well as various counts of base composition (Supplemental Table 3). The initiation classifier also included these features but only for the 100 bases after the TSS. Details on how we calculated each feature for each bin are available in Supplemental Methods.

We divided each chromosome into positive and negative examples for each classifier, where the initiation classifier compared examples of promoters to nonpromoter sequences, and the termination classifier compared the CPA sites of genes to gene bodies. We trained the classifiers on examples from half of the yeast chromosomes, and within these eight chromosomes, we left one chromosome out for evaluation of performance and optimization of the models. For each chromosome left out, we made four replicate forests, each with 50 trees. Thus, each classifier consisted of 1600 decision trees and was trained on half the genome.

### Creation and analysis of the combinatorial promoter library

We designed several hundred promoter segments encompassing the most important feature bins of the initiation classifier. We generated random DNA sequences, embedded TFBSs within each, and scored them by the initiation classifier in every possible combination. We selected those sequences that had relatively high initiation scores in multiple contexts for synthesis, and designed corresponding control sequences which were identical, but with the added TFBS perturbed. We also designed high and low G/C content promoter segments that either score highly or lowly, but into which we did not specifically add TFBSs. TFBSs were generated by sampling from the position frequency matrix of the corresponding factor using the frequencies as weights. The segments had 43, 48, and 41 different sequences in the −150:−80, −80:TSS, and TSS:+80 positions, respectively, and these had complementary overhangs at either end that ensured the segments would combine in the desired order and would yield, in theory, 86,688 different promoters.

We ordered the promoter parts as single-stranded oligonucleotides from Sigma-Aldrich. To generate double-stranded promoter parts, we phosphorylated and annealed the forward and reverse strands for each sequence. We then pooled and ligated these double-stranded promoter segments in bulk, purified and cloned the resulting promoters into an expression vector containing GFP, and transformed them into *E. coli*. We then isolated the resulting library and transformed it into yeast cells. We sorted the pooled yeast library by flow cytometry into six fluorescence bins and grew the sorted cells overnight. We then isolated DNA and amplified, barcoded, and sequenced the promoter DNA to determine the proportion of each promoter in each bin. We calculated the expression level of each promoter by weighting the probability of each promoter ending up in each bin by the average fluorescence of the bin.

## Data access

The data from this study have been submitted to the NCBI Gene Expression Omnibus (GEO; http://www.ncbi.nlm.nih.gov/geo/) under accession numbers GSE47004 and GSE48860. Supplemental and unprocessed data are also available at the author's website: http://hugheslab.ccbr.utoronto.ca/supplementary-data/transcription_model/.
